# Impact of Antenatal Corticosteroids on Cortisol and Glucose Homeostasis Levels in Preterm Neonates: A Meta-Analysis

**DOI:** 10.7759/cureus.74763

**Published:** 2024-11-29

**Authors:** Prerana S Bharadwaj, Srikrishna V Acharya

**Affiliations:** 1 Pediatrics, K S Hegde Medical Academy, Mangaluru, IND

**Keywords:** antenatal corticosteroids, blood-glucose, cortisol, hypoglycemia risk, preterm neonate

## Abstract

Antenatal corticosteroids (ACS) are widely used to reduce respiratory distress syndrome (RDS) in preterm neonates, enhancing neonatal outcomes. However, the potential effects of ACS on other aspects of neonatal health, such as cortisol levels and glucose regulation, remain a concern. This study examines whether ACS administration impacts cortisol and glucose homeostasis in preterm infants by analyzing data from 14 selected studies. Using a random-effects model, we found no significant impact of ACS on cortisol levels (mean differences (MD) 2.23, confidence interval (CI) 5.26 to -0.80) or blood glucose levels (standard mean differences (SMD) 0.18, CI 0.00 to 0.35). Additionally, there was no notable difference in hypoglycemia risk between groups receiving ACS and those unexposed (odds ratio (OR) 1.46, CI 0.99 to 2.17). Subgroup and sensitivity analyses reinforced these findings, underscoring their robustness, and risk-of-bias assessment confirmed a low risk across the included studies. Our findings support the safety of ACS for cortisol and glucose levels in preterm infants, affirming its continued use for lung development while recommending vigilant blood glucose monitoring to manage potential hypoglycemia. These results provide essential insights for neonatal care protocols, contributing to the overall welfare of premature infants.

## Introduction and background

Introduction

In recent years, notable advancements in neonatal care have greatly improved the outcomes for preterm neonates. One key factor in this progress has been the use of corticosteroids (antenatal corticosteroids, or ACS), which are commonly used to help fetal organs mature better and particularly benefit the development of the lungs. The positive effects of ACS in lowering the occurrence of respiratory distress syndrome (RDS) in premature babies are well documented and have played a crucial role in significantly reducing both mortality and health issues among neonates [1,2]. However, even though it's well known that ACS has positive effects on pulmonary outcomes, there's an increasing focus on exploring how giving ACS could affect other aspects of newborn health in general.

Significance of study

In particular, the potential impact of ACS on cortisol levels and glucose homeostasis in preterm neonates has emerged as an area of interest and concern [3,4]. On that note, this study is paramount due to its potential implications for neonatal care, where informed decision-making regarding ACS administration can significantly impact the health and well-being of preterm neonates. It is crucial to consider the long-term health consequences of ACS, and this meta-analysis aims to better understand the potential risks and benefits associated with its use.

Objective

This meta-analysis investigates the impact of ACS on cortisol and glucose homeostasis levels in preterm neonates.

Rationale

Studying cortisol is important because it is a steroid hormone that impacts different organ systems in the body. It helps neonates respond to stress, regulates blood pressure and metabolism, and hence impacts their growth. High levels of cortisol in neonates were associated with an increased incidence of intraventricular hemorrhage and impaired neurodevelopment [5]. Our investigation aims to explore the impact of ACS on cortisol levels.

In earlier studies, Uno et al. [6] and de Vries et al. [7] observed that repeated exposure to synthetic glucocorticoids (sGC) in rhesus monkeys and vervet monkeys resulted in elevated cortisol levels and lasting changes in stress responses in their offspring. Conversely, Weiss and Niemann [8] reported a diminished cortisol response in preterm infants exposed to glucocorticoids compared to their non-exposed counterparts. Similarly, Teramo et al. [9] found no significant difference in serum cortisol levels between newborns treated prenatally with betamethasone and those treated with a placebo, suggesting that betamethasone did not exert a sustained effect on fetal cortisol concentration in the studied groups. In contrast, Ryu et al. [10] suggested that ACS might increase cortisol levels. This diverse body of evidence underscores the intricate nature of the relationship between ACS exposure and cortisol levels in preterm neonates, emphasizing the necessity for a careful investigation of these dynamics.

Understanding the intricate connection between ACS and neonatal glucose regulation is essential for optimizing newborn health. Maintaining stable blood glucose levels in neonates is critical, and since ACS can influence maternal glucose levels, it may have significant implications for preterm infants [4,11]. Studies indicate an association between systemic steroids, including dexamethasone, and hypoglycemia [12]. Furthermore, corticosteroids, like dexamethasone, have been linked to metabolic complications affecting glucose homeostasis [13]. It's important to note that corticosteroid therapy, including ACS, can lead to maternal effects such as adrenal suppression and altered glucose tolerance [14]. Given this complex landscape, a targeted meta-analysis evaluating the impact of ACS on glucose homeostasis in preterm neonates becomes indispensable.

Hypothesis

Administering ACS to mothers of preterm neonates might not lead to consistent changes in cortisol levels or glucose homeostasis in their offspring.

## Review

Methods

Study Selection and Data Extraction

Study selection: Our hypothesis suggests that administering ACS to mothers of preterm neonates might lead to changes in cortisol levels and glucose homeostasis in their offspring. Unravelling the potential effects of ACS on these parameters is crucial for neonatal care and holds the potential to shape clinical practice guidelines.

Data extraction: Data from the included studies were extracted and organized in tabular format. The extracted data included baseline characteristics, such as first author, year of publication, country, study years, sample size, gestational age, type of steroid used, comparison groups, outcomes, and neonatal characteristics (e.g., mean birthweight, gestational age, cesarean delivery, and male sex). Furthermore, information on the doses of steroids administered, frequency of administration, time of administration, measurements of outcomes, and sampling times was documented in a separate table.

Data Source and Search Strategy

Based on the suggestions provided in the Preferred Reporting Items for Systematic Reviews and Meta-Analyses (PRISMA) 2020 [15] guidelines, the search encompassed English articles from the beginning up to August 2023. Various databases, such as PubMed, ScienceDirect, Embase, Medline, Cochrane, and Google Scholar, were meticulously searched. The exploration involved the use of keywords like "antenatal corticosteroid," "glucose," "cortisol," "betamethasone," "dexamethasone," "hypoglycemia," "glucose homeostasis," "preterm," and "neonate.”

Endpoints: The primary endpoints were blood glucose level and cortisol level. The secondary endpoints were hypoglycemia and the lowest blood glucose level at various sampling times.

Statistical Analysis

We applied a random-effects model for each outcome in the meta-analysis for statistical analysis. Our goal was to understand variations in the data, considering differences in participants, interventions, and study designs. The I² statistic was used to measure heterogeneity, with thresholds of 30%-60% indicating moderate heterogeneity, 50%-90% substantial heterogeneity, and 75%-100% considerable heterogeneity [16]. We have reported pooled odds ratios (OR) with corresponding 95% confidence intervals (CIs) for dichotomous outcomes. For continuous outcomes, pooled standard mean differences and mean differences were provided, each accompanied by their respective 95% CIs. Forest plots are included to represent the variation among the studies visually.

Results

**Figure 1:**
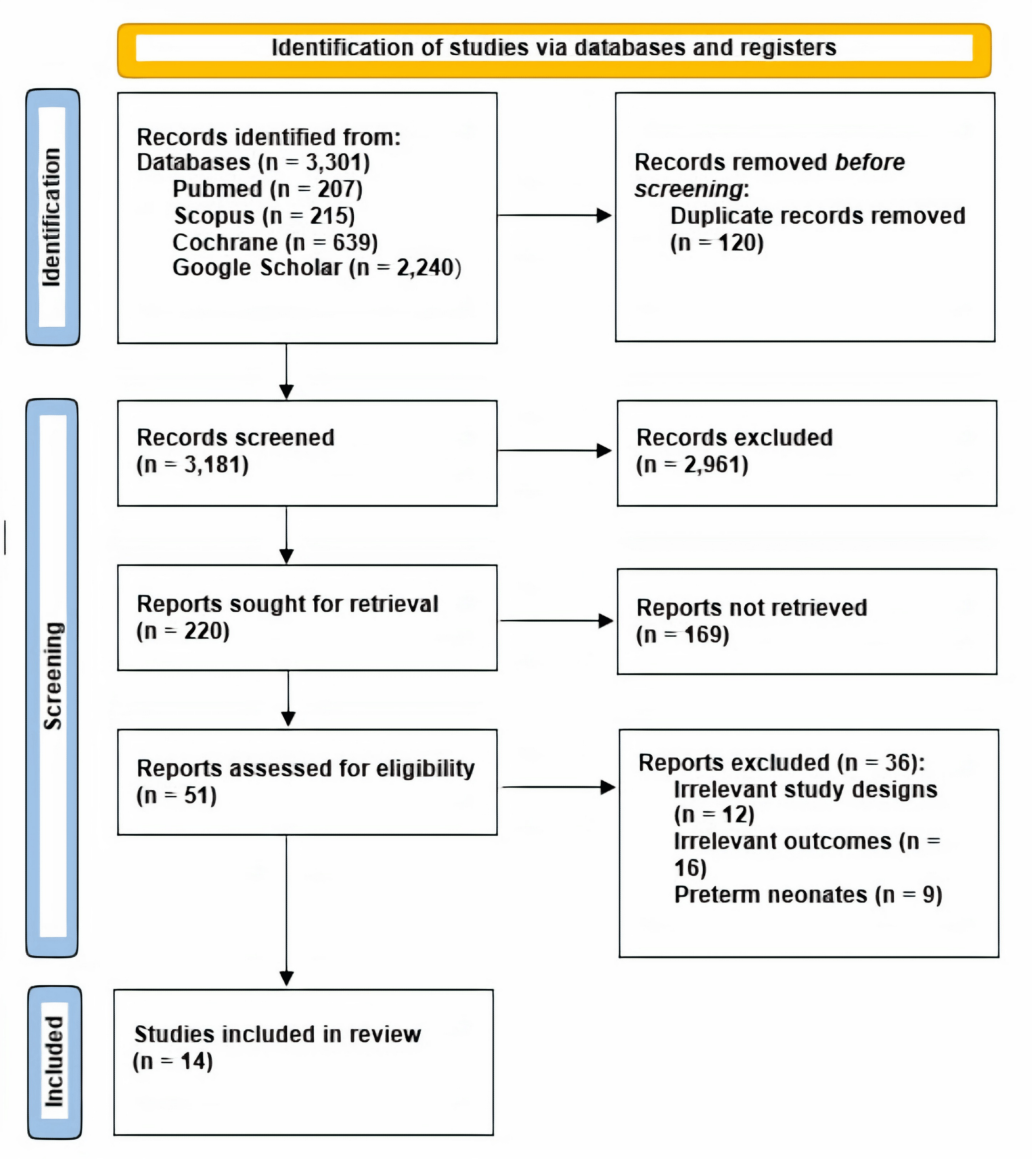
Preferred Reporting Items for Systematic Reviews and Meta-Analyses (PRISMA) flow chart summarizing the literature search

In the context of this meta-analysis, a total of 14 studies were examined, each with distinct characteristics. Of these, 13 were cohort studies, and one was a pilot study, all of which were included in the analysis. The flowchart summarizing the literature search following PRISMA guidelines is provided in Figure 1.

Impact of ACS on Cortisol, Blood Glucose, and Hypoglycemia

This section presents the meta-analysis findings on how ACS affect cortisol levels, blood glucose, and hypoglycemia in preterm neonates. The analysis compares different groups and treatment courses, including the impact of administering ACS compared to no treatment; the outcomes between neonates whose mothers received a full course versus a partial course of ACS; the differences between receiving a partial ACS course and no ACS at all; and the comparison of a complete ACS course with no ACS administration. Additionally, a subgroup analysis was conducted to determine whether specific populations are more affected by ACS, and a sensitivity analysis was performed to examine the robustness of results by excluding individual studies and observing their influence on overall outcomes.

ACS Exposure Group and No ACS Exposure Group

**Figure 2:**
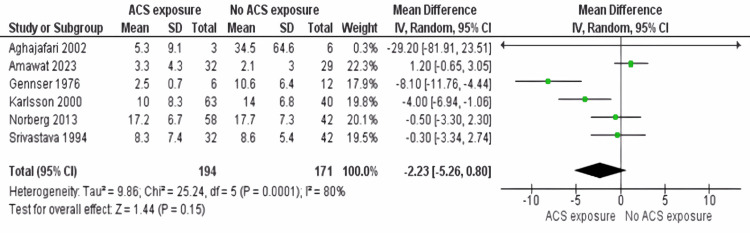
Cortisol level outcome between ACS exposure group and no ACS exposure group ACS: antenatal corticosteroids [17-22]

Cortisol: In a meta-analysis involving six papers [17-22], we studied how administering ACS affects cortisol levels in individuals during pregnancy. When comparing results between those who received ACS and those who did not receive it, we did not find a connection (MD -2.23; 95% CI -5.26 to 0.80; p = 0.15, see Figure 2). Significant differences were noticed (I^2^ = 80%, p = 0.15), showing diverse results amongst all research studies considered in this analysis. It's important to mention that this analysis is part of a study that also looks into outcomes related to cortisol levels, blood glucose, and hypoglycemia.

**Figure 3:**
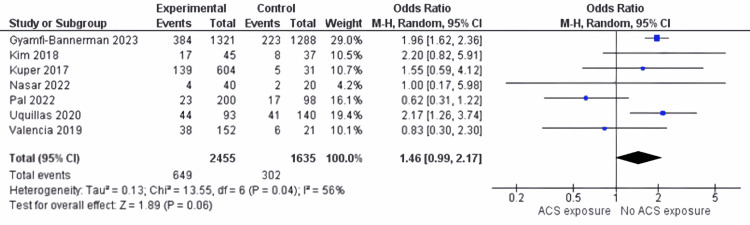
Hypoglycemia outcome between ACS exposure group and no ACS exposure group ACS: antenatal corticosteroids [23-29]

Hypoglycemia: In our review of seven papers [23-29], we delved into how ACS administration relates to hypoglycemia risk assessment. The findings showed a non-significant difference (OR 1.46: 95% CI 0.99 to 2.17; p = 0.06, see Figure 3) but demonstrated substantial heterogeneity (I^2^ = 56% (p = 0.04)).

**Figure 4:**
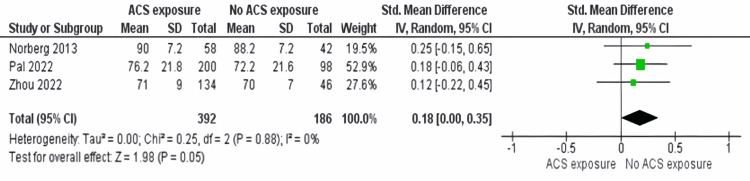
Blood glucose outcome between ACS exposure group and no ACS exposure group ACS: antenatal corticosteroids [21,27,4]

Blood glucose: Our meta-analysis, which included three papers [4,21,2], is aimed to assess how ACS administration relates to blood glucose levels. The analysis showed no significant association with low heterogeneity (I^2^ = 0%, p = 0.88, see Figure 4).

ACS Complete Course and ACS Partial Course

**Figure 5:**
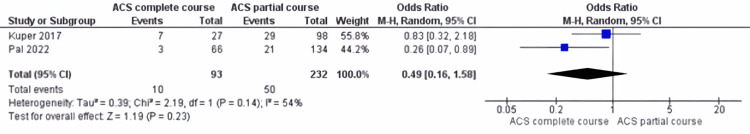
Hypoglycemia outcome between ACS complete course group and ACS partial course group ACS: antenatal corticosteroids [25,27]

Hypoglycemia: After comparing incomplete courses and partial courses of ACS treatment for hypoglycemia, no significant differences were observed (OR 0.49: 95% CI 0.16 to 1.58; p = 0.23, see Figure 5). However, substantial heterogeneity was present (I^2^ = 54% (p = 0.14)).

**Figure 6:**
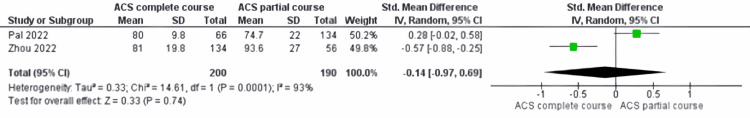
Blood glucose outcome between ACS complete course group and ACS partial course group ACS: antenatal corticosteroids [27,4]

Blood glucose: Moreover, in terms of blood glucose levels, no notable variation was observed (standard mean differences (SMD) -0.14: 95% CI -0.97 to 0.69; p = 0.74, see Figure 6), with considerable heterogeneity (I^2^ = 93% (p<0.0001)).

ACS Partial Course and No ACS Course

**Figure 7:**
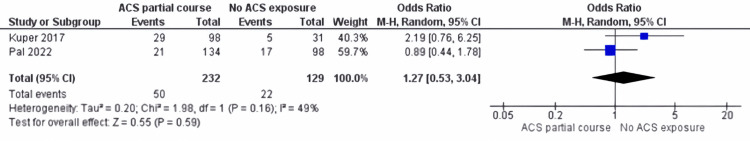
Hypoglycemia outcome between ACS partial course group and no ACS group ACS: antenatal corticosteroids [25,27]

Hypoglycemia: Furthermore, no significant association was observed between the ACS partial course and the no ACS exposure group in hypoglycemia (OR 2.37: 95% CI 0.53 to 3.04; p = 0.59, see Figure 7), with moderate heterogeneity (I^2^ = 49% (p = 0.59)).

ACS Complete Course and No ACS Course

**Figure 8:**
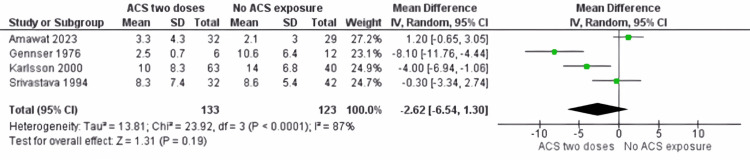
Cortisol outcome between ACS complete course group and no ACS group ACS: antenatal corticosteroids [18-20,22]

Cortisol: The comparison between the complete ACS administration group and the no ACS group showed no significant difference in cortisol levels (MD -2.62: 95% CI -6.54 to 1.30; p = 0.19, see Figure 8), with considerable heterogeneity (I^2^ = 87% (p < 0.0001)).

**Figure 9:**
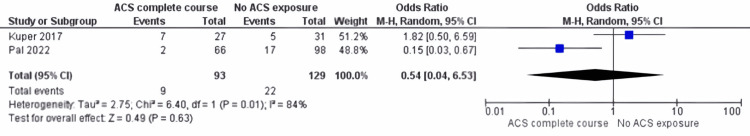
Hypoglycemia outcome between ACS complete course group and no ACS group ACS: antenatal corticosteroids [25,27]

Hypoglycemia: Additionally, in this same comparison, no significant association was found in hypoglycemia (OR 0.57: 95% CI 0.04 to 6.53; p = 0.63, see Figure 9), with considerable heterogeneity (I^2^ = 84% (p = 0.01)).

**Figure 10:**
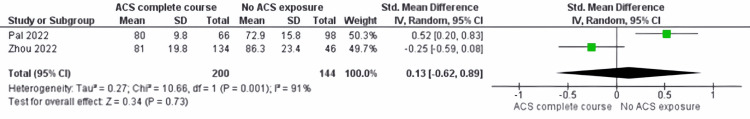
Blood glucose outcome between ACS complete course group and no ACS group ACS: antenatal corticosteroids [27,4]

Blood glucose: The study did not find any association with blood glucose levels when comparing those who had the complete ACS course and those with no ACS (SMD 0.13: 95% CI -0.62 to 0.89; p = 0.73, see Figure 10). It is worth mentioning that there was considerable heterogeneity (I^2^ = 91% (p = 0.001)).

Subgroup Analyses

The subgroup analyses aim to gain a deeper understanding of the effects of ACS administration on preterm neonates. Specific subgroups are examined by considering different timeframes and delivery scenarios. This exploration aims to identify potential variations in ACS's impact on hypoglycemia occurrence and cortisol levels, providing further insights into its influence on neonatal health. The detailed interpretations that follow highlight the specific dynamics observed in the subgroups.

**Figure 11:**
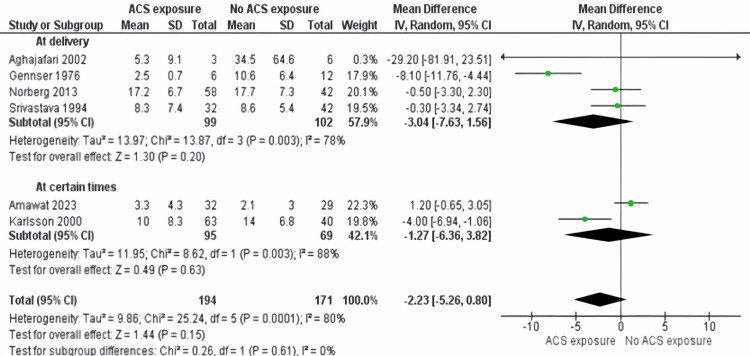
Cortisol outcome with subgroup based on the difference of time sampling ACS: antenatal corticosteroids At delivery: [17,19,21,22]; At certain times: [18,20]

Cortisol: The subgroup analyses uncover notable insights into cortisol levels in preterm neonates. The findings reveal no significant differences in cortisol levels between the two groups, whether at delivery or at specific time points, as demonstrated in Figure 11.

At delivery: The analyses indicated no significant difference between the two groups in cortisol levels at the time of delivery (SMD -3.04: 95% CI -7.83 to 1.56; p = 0.20). Considerable heterogeneity was observed (I^2^ = 76% (p = 0.003)), indicating variations in cortisol outcomes among the included studies.

At certain times: No significant difference was found between the two groups concerning cortisol levels at specific time points (SMD -1.27: 95% CI -6.36 to 3.82; p = 0.63). Considerable heterogeneity was noted (I^2^ = 88% (p = 0.003)), emphasizing the diversity in cortisol measurements during these selected instances.

**Figure 12:**
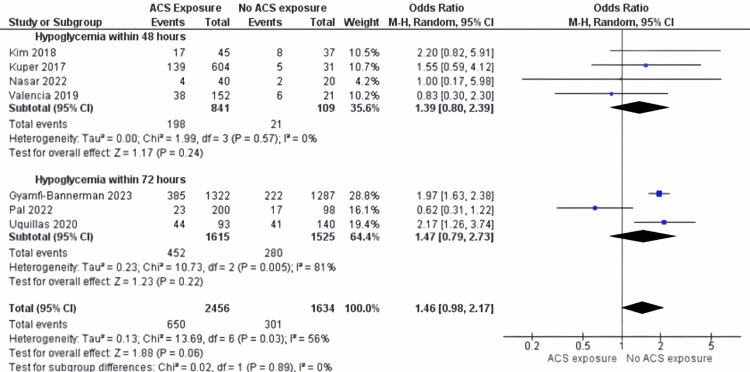
Hypoglycemia outcome with subgroup based on the difference of time sampling ACS: antenatal corticosteroids Hypoglycemia within 48 hours: [24-26,29]; Hypoglycemia within 72 hours: [23,27,28]

Hypoglycemia: The subgroup analyses provide valuable insights into the incidence of hypoglycemia in preterm neonates. The results indicate no significant differences between the two groups within 48 hours and 72 hours, as detailed in Figure 12.

Within 48 hours: The analyses revealed no significant difference between the two groups regarding the incidence of hypoglycemia (OR 1.39: 95% CI 0.80 to 2.39; p = 0.24). Heterogeneity was not significant (I^2^ = 0% (p = 0.57)), indicating consistent results across studies within this timeframe.

Within 72 hours: No significant difference was observed between the two groups regarding hypoglycemia occurrence (OR 1.47: 95% CI 0.79 to 2.73; p = 0.22). However, there was considerable heterogeneity (I^2^ = 81% (p = 0.005)) suggesting variability in study outcomes within this extended timeframe.

These findings collectively enhance our understanding of the impact of ACS administration on cortisol levels and hypoglycemia, taking into account various timeframes and contexts. The observed heterogeneity emphasizes the importance of careful interpretation and highlights potential variations across studies, suggesting that individual factors may influence outcomes in preterm neonates.

Sensitivity Analyses of Between-Study Heterogeneity

Sensitivity analysis is a crucial step in the meta-analysis, aimed at assessing the robustness of overall findings against potential influential factors or variations in study inclusion/exclusion criteria. Identifying these sources requires a thorough, case-by-case examination. This method involves systematically excluding individual studies one at a time to evaluate their impact on overall results and the level of heterogeneity between studies [30]. By doing so, sensitivity analysis provides valuable insights into the influence of specific studies on the summary effect and heterogeneity. This approach is essential for ensuring the reliability and validity of meta-analytic conclusions.

Sensitivity analyses were systematically performed for each outcome of cortisol levels, blood glucose levels, and hypoglycemia by excluding individual studies one at a time to assess their impact on the overall effect size and heterogeneity. The exclusion of any single study did not significantly alter the findings, confirming the robustness and reliability of the results across all measured outcomes.

Sensitivity analyses - cortisol: The results in Table 1 demonstrate that excluding individual studies had minimal impact on the overall effect of ACS on cortisol levels. The mean differences remained non-significant across all exclusions, and while heterogeneity fluctuated, it did not drastically change the overall interpretation. This consistency underscores the stability of our findings regarding cortisol levels in preterm neonates.

**Table 1:** Sensitivity analyses of between-study heterogeneity for cortisol levels

Study excluded	Mean Difference (95% CI)	p-value (effect)	Heterogeneity	p-value (heterogeneity)
Alghajafari 2002 [17]	-2.14 (-5.18, 0.89)	0.17	I^2^= 83%	<0.0001
Amawat 2023 [18]	-3.20 (-6.51, 0.11)	0.06	I^2^= 73%	0.005
Gennser 1976 [19]	-0.80 (-3.03, 1.43)	0.48	I^2^= 59%	0.04
Karlsson 2000 [20]	-1.82 (-5.35, 1.70)	0.31	I^2^= 81%	0.0003
Norberg 2013 [21]	-2.76 (-6.68, 1.15)	0.17	I^2^= 84%	<0.0001
Srivastava 1994 [22]	-2.78 (-6.61, 1.04)	0.15	I^2^= 84%	<0.0001

Sensitivity analyses - hypoglycemia: The inclusion or exclusion of specific studies notably influenced the relationship between ACS administration and hypoglycemia. As shown in Table 2, when studies by Pal et al. [27] and Valencia and Rojas [29] were excluded, significant changes were observed. In these cases, the ACS group demonstrated a significant association with hypoglycemia compared to the "No ACS" group, with ORs of 1.91 (95% CI 1.62 to 2.26; p < 0.00001) and 1.56 (95% CI 1.04 to 2.34; p = 0.03), respectively. In contrast, excluding other studies did not substantially alter the overall outcome of the meta-analysis.

**Table 2:** Sensitivity analyses of between-study heterogeneity for hypoglycemia outcome The asterisk (*) indicates that excluding the studies by Pal et al. [27] and Valencia and Rojas [29] resulted in significant changes in the meta-analysis outcomes.

Study excluded	Odds ratio (95% CI)	p-value (effect)	Heterogeneity	p-value (heterogeneity)
Gyamfi Bannerman 2023 [23]	1.29 (0.77, 2.15)	0.33	I^2^= 50%	0.08
Kim 2018 [24]	1.37 (0.88, 2.14)	0.16	I^2^= 63%	0.02
Kuper 2017 [25]	1.43 (0.91, 2.24)	0.12	I^2^= 63%	0.02
Nasar 2022 [26]	1.48 (0.98, 2.24)	0.07	I^2^= 62%	0.02
Pal 2022* [27]	1.91 (1.62, 2.26)	<0.00001	I^2^= 0%	0.61
Uquillas 2020 [28]	1.30 (0.78, 2.15)	0.32	I^2^= 62%	0.02
Valencia 2019* [29]	1.56 (1.04, 2.34)	0.03	I^2^= 56%	0.05

A focused analysis was conducted to further explore these findings, considering only Pal et al. [27] and Valencia and Rojas [29] studies.

Focused analysis of Pal et al. [27] and Valencia and Rojas [29] studies: A focused analysis was conducted exclusively on the Pal et al. [27] and Valencia and Rojas [29] studies to examine their specific influence on the overall associations. This targeted approach evaluates the internal consistency of these studies and determines whether they consistently contributed to the significant association with hypoglycemia observed in the sensitivity analysis.

**Figure 13:**
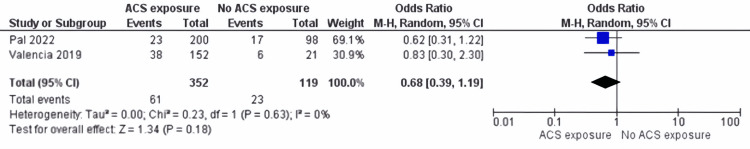
Focused sensitivity analysis of hypoglycemia outcome ACS: antenatal corticosteroids [27,29]

As illustrated in Figure 13, the results from this separate meta-analysis, which focused solely on Pal et al. [27] and Valencia and Rojas [29], revealed no statistically significant difference between the two groups (p = 0.18). Although these studies showed a notable impact during the sensitivity analysis, their exclusion did not appear to be the primary driver of the overall effect. Had they consistently contributed to a significant association with hypoglycemia, it would have bolstered the evidence for the observed outcome. However, the absence of statistical significance suggests that their influence aligns with the overall trend and does not deviate significantly from the broader meta-analysis findings.

Sensitivity analyses - blood glucose: The sensitivity analyses, conducted by excluding individual studies one at a time, showed that removing any single study did not significantly change the overall findings. This confirms the robustness and reliability of the meta-analysis regarding blood glucose levels. As shown in Table 3, the results remained consistent across the excluded studies, with minimal heterogeneity observed (I² = 0%).

**Table 3:** Sensitivity analyses - blood glucose SMD: standard mean differences

Study excluded	SMD (95%CI)	p-value	Heterogeneity	p-value (heterogeneity)
Norberg 2013 [21]	0.16 (-0.04, 0.36)	0.11	I² = 0%	0.75
Pal 2022 [27]	0.17 (-0.09, 0.43)	0.19	I² = 0%	0.62
Zhou 2022 [4]	0.20 (-0.01, 0.41)	0.06	I² = 0%	0.79

Summary of sensitivity analysis for cortisol, hypoglycemia, and blood glucose: The comprehensive sensitivity analyses performed across the outcomes of hypoglycemia, blood glucose, and cortisol reaffirmed the robustness of the overall meta-analysis. While certain studies notably influenced the association between ACS administration and hypoglycemia, the findings related to blood glucose and cortisol levels remained stable and unaffected by the exclusion of individual studies. This consistency in results enhances the reliability of the meta-analysis and strengthens the confidence in the validity of the reported associations across all outcomes.

Risk of Bias Analysis

**Figure 14:**
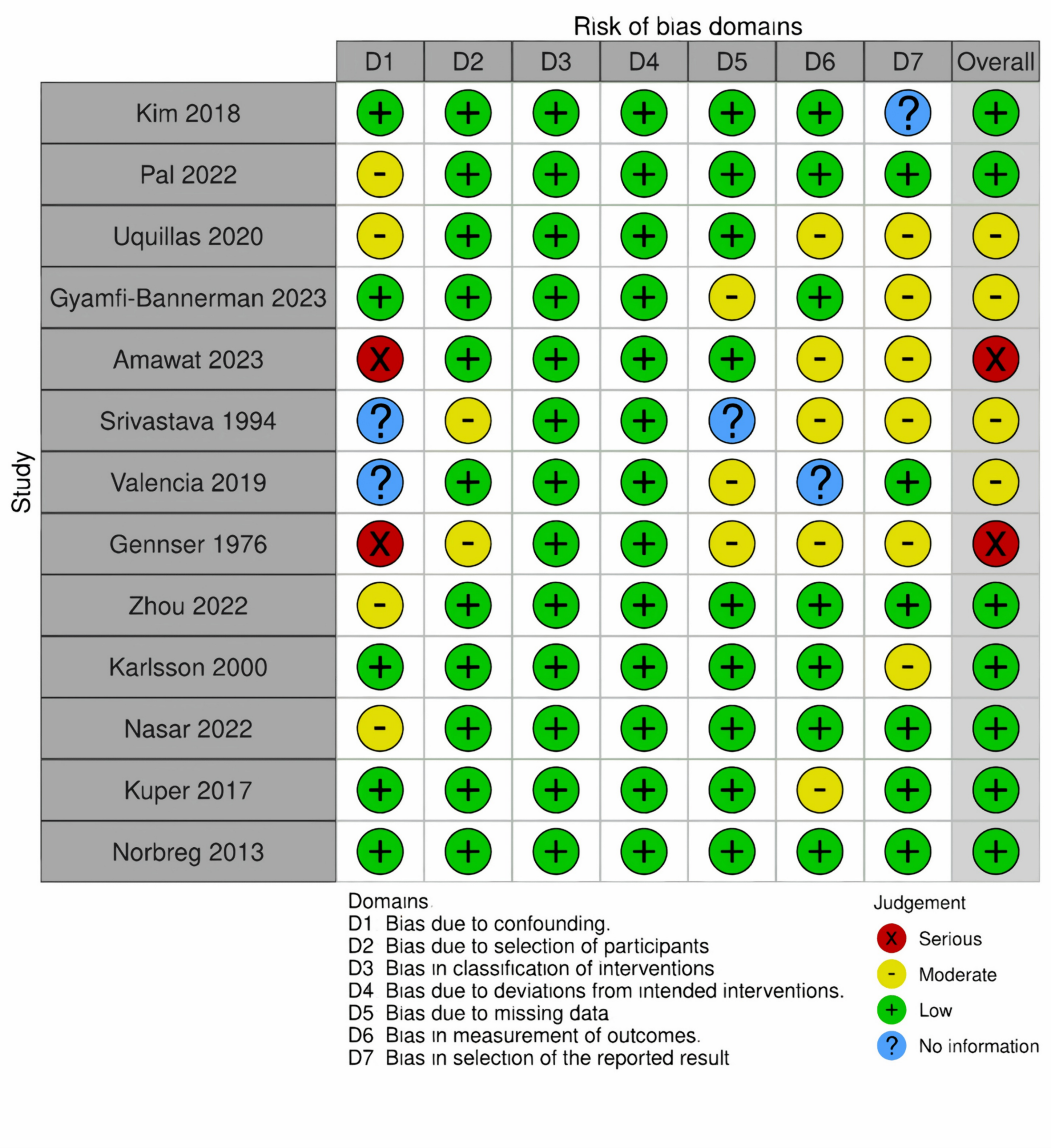
Studies quality assessment using ROBINS-I ROBINS-I: Risk of Bias In Non-randomized Studies - of Interventions [24,27,28,23,18,22,29,19,4,20,26,25,21]

**Figure 15:**
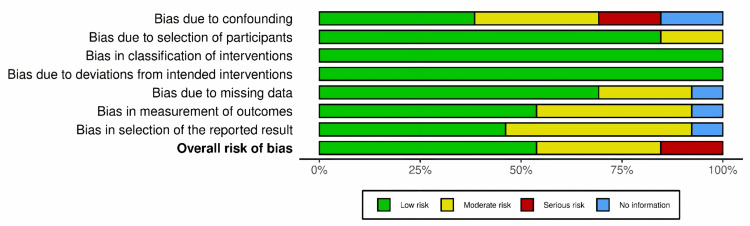
Overall risk of bias - summary of domains

In assessing the quality of the included studies, the Risk of Bias In Non-randomized Studies - of Interventions (ROBINS-I) tool was applied to evaluate 13 studies. Notably, one study [17], classified as a pilot study, posed challenges in risk-of-bias assessment due to the limitations of tools such as RoB v2, ROBINS-I, or NoS. The ROBINS-I assessment, detailed in Figure 14, illustrates the risk of bias across seven key domains (D1 to D7) for each study, with the majority demonstrating a low risk of bias. Figure 15 provides an overview of the overall risk of bias, visualized through a bar graph that categorizes the studies into different categories of risk across these domains. Together, these figures emphasize that most studies maintained a low to moderate risk of bias, enhancing the reliability of the overall findings.

Discussion

Limitations and Methodological Rigor

This meta-analysis is subject to several limitations that should be carefully considered. The studies included were conducted across a variety of clinical settings, which introduced differences in geographic locations, population sizes, and participant characteristics such as age and weight. These factors contribute to heterogeneity that may affect the generalizability of the findings.

Another key challenge arose from the variations in how outcomes were measured and reported across studies. This inconsistency limited the extent to which the data could be fully synthesized and rigorously analyzed. Furthermore, the presence of studies with lower methodological quality, including those with serious risk of bias, reduced the overall strength of the analysis.

Despite these limitations, the robustness of the findings was reinforced through methodological safeguards and thorough analyses.

Addressing Limitations and Ensuring Rigor

Several critical steps were implemented to strengthen this meta-analysis's methodological integrity. A comprehensive sensitivity analysis was conducted, systematically excluding individual studies to assess their influence on the overall results. This approach helped identify potential biases and confirm the stability of the findings. Subgroup analyses were also carried out to examine the role of specific factors, offering a deeper understanding of variations across different populations and contexts. Furthermore, all included studies underwent a thorough risk-of-bias assessment, allowing for a careful evaluation of study quality. Together, these efforts enhance the transparency and reliability of the meta-analysis, ensuring a balanced and credible interpretation of the findings.

Interpretation and Implications for Clinical Practice

This meta-analysis indicates that the administration of ACS does not significantly influence cortisol levels or blood glucose in preterm neonates. Stable blood glucose, crucial for preventing hypoglycemia-linked adverse neurodevelopmental outcomes, remains unaffected by ACS administration, offering reassurance that this intervention does not disrupt glucose homeostasis. Additionally, no statistically significant increase in hypoglycemia risk was observed between the ACS and control groups. Despite these reassuring findings, continued monitoring of neonates exposed to ACS is essential, given its potential implications for neonatal health. The insights regarding cortisol and blood glucose levels can inform clinical guidelines, emphasizing the need for vigilant monitoring to support proactive management and prevent complications in neonates receiving ACS.

The decision-making process for healthcare providers and expectant parents regarding ACS use in preterm labor management is critical. The results affirm the safety profile of ACS, reinforcing its role in supporting informed clinical decisions. Parents can confidently discuss the benefits and potential risks with healthcare providers, knowing that ACS is unlikely to adversely affect cortisol or blood glucose levels. Understanding these impacts is key to optimizing care for preterm neonates, enabling healthcare providers to anticipate and address challenges efficiently, ultimately improving neonatal outcomes.

## Conclusions

This review meticulously examined the impact of ACS administration on key health outcomes in preterm neonates, including cortisol levels, glucose homeostasis, and the risk of hypoglycemia. The findings suggest that administering ACS for fetal lung development is unlikely to negatively influence cortisol levels, providing a reassuring outcome for clinicians and supporting the continued use of ACS in preventing respiratory complications in preterm births. Moreover, ACS administration did not result in a statistically significant difference in blood glucose levels, a critical factor in preventing hypoglycemia, which is linked to long-term neurodevelopmental issues. This highlights that ACS does not compromise glucose homeostasis, further reinforcing its safety in clinical practice. Although no significant difference was found in hypoglycemia risk between neonates exposed to ACS and those unexposed, the study emphasizes the necessity of continuous monitoring in neonates to mitigate any potential risks and ensure optimal neonatal care.

In conclusion, the evidence supports the safety of ACS administration in preterm neonates, particularly regarding its neutral effects on cortisol and blood glucose. Clinicians may consider ACS as a safe intervention to support neonatal outcomes, with minimal concern for significant metabolic disruptions, based on current evidence. However, continued monitoring is recommended to promptly address any potential complications and support optimal neonatal care.
